# Gastric herpes simplex virus type 1 infection is associated with functional gastrointestinal disorders in the presence and absence of comorbid fibromyalgia: a pilot case–control study

**DOI:** 10.1007/s15010-022-01823-w

**Published:** 2022-04-21

**Authors:** Carol Duffy, William L. Pridgen, Richard J. Whitley

**Affiliations:** 1 The Department of Biological Sciences, University of Alabama, Tuscaloosa, AL USA; 2 Tuscaloosa Surgical Associates, Tuscaloosa, AL USA; 3 Division of Pediatric Infectious Diseases, Department of Pediatrics, Heersink School of Medicine, University of Alabama at Birmingham, Birmingham, AL USA

**Keywords:** Herpes simplex virus, Functional gastrointestinal disorders, Fibromyalgia, Somatic symptom disorder, Irritable bowel syndrome

## Abstract

**Purpose:**

Animal studies have linked gastric herpesvirus infections to symptoms associated with functional gastrointestinal disorders (FGIDs). Herpesviruses have also been hypothesized to contribute to fibromyalgia (FM), a chronic pain syndrome frequently comorbid with FGIDs. The purpose of this study was to compare the prevalence of gastric herpesvirus infection in patients with FGIDs, with and without comorbid FM, to that of controls.

**Methods:**

For this pilot case–control study, we enrolled 30 patients who met both the Rome IV diagnostic criteria for one or more FGIDs and the American College of Rheumatology 2010 criteria for FM, 15 patients with one or more FGIDs without comorbid FM, and 15 control patients. Following endoscopic examination, gastric biopsies were analyzed for herpesvirus DNA and protein, *Helicobacter pylori* infection, and histological evidence of gastritis. Importantly, the viral nonstructural protein ICP8 was used as a marker to differentiate cell-associated actively replicating virus from latent infection and/or free virus passing through the GI tract.

**Results:**

Gastric herpes simplex virus type 1 (HSV-1) infection, as indicated by ICP8 presence, was significantly associated with FGIDs in the presence (OR 70.00, 95% CI 7.42–660.50; *P* < .001) and absence (OR 38.50, 95% CI 3.75–395.40; *P* < .001) of comorbid FM. Neither histological gastritis nor *H. pylori* infection were found to be associated with FGIDs or FM.

**Conclusions:**

HSV-1 infection was identified in gastric mucosal biopsies from patients with diverse FGIDs, with and without comorbid FM. Larger, multi-center studies investigating the prevalence of this association are warranted.

**Supplementary Information:**

The online version contains supplementary material available at 10.1007/s15010-022-01823-w.

## Background

Functional gastrointestinal disorders (FGIDs), a group of conditions characterized by chronic gastrointestinal (GI) symptoms, comprise a significant social and economic burden. With a worldwide prevalence of 40%, FGIDs account for a large percentage of primary and secondary health-provider workload, medication usage, work absenteeism, and reduced quality of life [2–6]. Diagnoses of FGIDs rely on symptom-based criteria outlined by the Rome Foundation [7]. The current classification system (Rome IV) divides FGIDs into 33 adult and 20 pediatric disorders [8, 9]. Historically defined as GI conditions with no organic basis, FGIDs are now believed to arise from neurogastroenterological disturbances and alterations in gut-brain communication [10–14]. Physiological factors including abnormal motility, visceral hypersensitivity, barrier dysfunction, immune dysregulation, and altered microbiome composition are believed to contribute to the development of FGIDs [11, 15]. Psychosocial factors, which influence GI tract physiology through the gut-brain axis, may also contribute to FGID development and persistence [11].

Patients frequently present with multiple FGIDs affecting different regions of the GI tract [4, 16–20]. Mechanistic hypotheses for overlapping FGIDs include a common pathogenesis that alters gut-brain interactions, activates the peripheral and enteric immune systems, and causes neuroendocrine dysregulation [17]. Parasitic, bacterial, and viral infections have been proposed as etiologic agents for overlapping FGIDs [17, 21–23]. Human herpesviruses are viable etiologic candidates as they establish persistent infections, affect the relevant pathophysiological pathways, and readily infect the GI tract [24–34].

A hallmark of all herpesviruses is their ability to establish life-long quiescent infections, termed latency, in one or more cell types. Although several herpesviruses infect the GI mucosa [24–34], only herpes simplex virus types 1 and 2 (HSV-1/-2) and varicella zoster virus are known to establish latency in ganglionic neurons that innervate the GI tract [35–41]. These neurotropic herpesviruses initially infect and replicate within mucosal epithelial cells. Virions produced during this active, lytic infection spread to nearby cells and invade ganglionic neurons where latency is established. Reactivation from latency results in the production of virions that spread to the innervated gut mucosa and commence another round of lytic infection. Neurotropic herpesviruses also spread trans-synaptically and are known to invade, replicate, and spread within the central, peripheral, and enteric nervous systems [35–46].

Several animal studies have linked herpesvirus infections to symptoms associated with functional gastrointestinal disorders (FGIDs) [37, 42, 44–46]. For example, Brun et al. showed intranasal and intragastric infection of rats with HSV-1 led to the establishment of latency in myenteric ganglia of the enteric nervous system and resulted in gut dysmotility [37]. Follow-up studies showed HSV-1 infection of rat enteric neurons can trigger gut dysfunction through mechanisms involving macrophage recruitment and modulation of the adenosinergic system [42, 45]. These studies suggest neurotropic herpesvirus infections can spread within the gut mucosal epithelium and gut–brain neural pathways and may potentially contribute to the symptoms of FGIDs.

Herpesviruses have also been hypothesized to contribute to fibromyalgia (FM), a syndrome characterized by chronic widespread pain, fatigue, and cognitive dysfunction. In a previous pilot clinical study, a drug combination targeting herpesvirus reactivation and lytic replication was safe and efficacious in the treatment of FM [47]. Irritable bowel syndrome (IBS) and other FGIDs are often comorbid with FM [48–50]. Because many patients with FGIDs experience overlapping upper and lower GI symptoms, we hypothesized gastric infection and spread of neurotropic herpesviruses within relevant neural pathways might contribute to the symptoms of IBS and other FGIDs. The aim of this study was to assess the prevalence of gastric herpesvirus infection in patients with FGIDs with and without comorbid FM. We hypothesized actively replicating herpesviruses would be more prevalent in gastric mucosal tissue from patients with these functional disorders than in that of controls.

## Methods

### Study design and participants

A pilot case–control study was performed at the University of Alabama in conjunction with Tuscaloosa Surgical Associates (Tuscaloosa, AL, USA), DCH Regional Medical Center (Tuscaloosa, AL, USA), Northport Medical Center (Northport, AL, USA), and Northport Surgical Center (Northport, AL, USA). The study was approved by the Institutional Review Boards overseeing these sites, and all subjects provided written informed consent prior to enrollment.

Study participants were recruited from male and female patients under the care of Tuscaloosa Surgical Associates (TSA), 19–79 years of age, who presented with clinical indications requiring upper endoscopy and who were not taking medications for HSV-1 or *H. pylori*. To identify potential differences between patients with and without comorbid FM, two case groups and one control group were enrolled. Group 1 was composed of 30 patients who met both the Rome IV criteria for one or more FGIDs and the American College of Rheumatology 2010 diagnostic criteria for FM [8, 51]. Group 2 was composed of 15 patients who met the Rome IV criteria for one or more FGIDs but without FM. The control group (Group 3) was composed of 15 patients who did not meet the diagnostic criteria for FGIDs, FM, or other somatic symptom disorders, but required upper endoscopy as a diagnostic for GI bleed (5 patients), ongoing anemia (2 patients), or hiatal hernia (1 patient); for screening prior to bariatric surgery (1 patient) or for GI cancer (3 patients); or as a follow-up to small bowel obstruction (1 patient) or GI surgery (2 patients).

### Endoscopic examination, biopsy collection, and histology

Endoscopy procedures on all subjects were performed by TSA using an Olympus EVIS EXERA II endoscopy system. During each procedure, the entire upper GI tract surface was inspected. Gastric biopsies 1–2 mm in size were captured using the cold biopsy technique. Biopsies were taken from regions of the antrum of the stomach showing erythema, streaking, or ulceration when present. Biopsy specimens were transferred to cryovials containing phosphate-buffered saline (PBS). Biopsy specimens from each subject were divided; part was used for histological examination, and part was frozen at − 20 °C for DNA and protein analyses. Histology specimens were prepared according to routine clinical protocols and interpreted by clinical pathologists at DCH Regional Medical Center who were blinded to the corresponding study groups. *H. pylori* was identified by Giemsa staining. Histologically identified gastritis was diagnosed and classified by the experienced pathologists at DCH according to the updated Sydney System [52–54].

### Protein and DNA sample preparation

Blinded gastric biopsy specimens frozen in 0.5 ml PBS were thawed and disrupted by probe sonication. For protein analyses, 100 μl was mixed with 50 μl 3X SDS-PAGE sample buffer (187.5 mM Tris–HCl [pH 7.0], 6% w/v SDS, 0.125 M dithiothreitol, 30% glycerol, 0.03% w/v bromophenol blue), denatured for 5 min at 100 °C, and stored at − 20 °C until use in immunoblot assays. For positive and negative controls, Vero cells (CCL-81, ATCC) were cultured at 37 °C in Dulbecco’s modified Eagle’s medium supplemented with 4.0 mM L-glutamine, 4.5 g/liter glucose, 125 units/ml penicillin, 0.125 mg/ml streptomycin, and 10% newborn calf serum. Confluent cell monolayers were either mock-infected or infected with HSV-1 strain F [55] at a multiplicity of infection (MOI) of 5 plaque-forming units (PFU) per cell and incubated at 37 °C. Cells were collected 18 h post-infection (hpi) and pelleted by centrifugation. Cell pellets were resuspended in SDS-PAGE sample buffer, denatured for 5 min at 100 °C, and stored at − 20 °C.

For DNA analyses, the remaining 400-μl sonicated sample from each biopsy was mixed with 2.5 μl 20 mg/ml Proteinase K and 5.0 μl 10% SDS and incubated for 18 h at 37 °C. Nucleic acids were isolated via phenol–chloroform–isoamyl alcohol extractions and ethanol precipitation [56]. Air-dried pelleted nucleic acids were resuspended in 50 μl sterile Milli-Q H_2_O. For positive and negative controls, confluent Vero cell monolayers were either mock-infected or infected with HSV-1 (strain F) at an MOI of 0.01 PFU per cell at 37 °C. Cells were collected at 18 hpi, pelleted by centrifugation, resuspended in PBS, and disrupted by probe sonication. Nucleic acids were purified via phenol–chloroform–isoamyl alcohol extractions and ethanol precipitation. The purity and concentration of isolated nucleic acids were determined by NanoDrop spectrophotometry (Thermo Scientific, Waltham, Massachusetts, USA).

### Nested PCR and DNA sequencing

Herpesvirus DNA was amplified by nested PCR using degenerate consensus primers according to an established protocol [57]. The published primers anneal to conserved sequences within the herpesvirus DNA polymerase gene, allowing PCR amplification of genomic DNA from any herpesvirus [57]. The annealed primers flank a non-conserved region of the herpesvirus DNA polymerase gene; thus, the particular herpesvirus(es) present in the template can be identified by sequencing the resulting PCR product. The nested PCR was performed in two rounds; 0.5 μg DNA isolated from biopsy specimens or cultured cells was used as the template for 50-μl round one reactions, whereas 1.0 μl of the round one PCR product was used as the template for 50-μl round-two reactions. Reactions containing template DNA isolated from Vero cells infected with HSV-1 and mock-infected Vero cells served as positive and negative controls for DNA isolation, PCR amplification, and instrument contamination. No-template PCR reactions were performed as additional controls for reagent contamination.

Round two PCR products were visualized with ethidium bromide staining following 1% agarose gel electrophoresis. PCR products were gel purified using the QIAquick Gel Extraction Kit (QIAGEN, Inc., Hilden, Germany) according to the manufacturer’s instructions. Automated Sanger sequencing using the round-two PCR primers was performed by Eurofins Genomics, Inc. (Louisville, Kentucky, USA). Sequence alignments were performed using the standard nucleotide Basic Local Alignment Search Tool (BLASTN) provided by the National Center for Biotechnology Information (NCBI).

### Immunoblot analyses

Denatured biopsy specimens and cultured cell lysates were separated by 12% SDS-PAGE and transferred to nitrocellulose membranes. Membranes were blocked with 5% skim milk in PBS then incubated with mouse anti-HSV-1 ICP8 antibody (ab20194, Abcam, Cambridge, UK). Membranes were washed extensively and incubated with anti-mouse IgG secondary antibody conjugated to horseradish peroxidase (NA9310V, GE Healthcare Biosciences, Piscataway, New Jersey, USA). Membranes were again washed extensively, incubated in Pierce ECL Western Blotting Substrate (Thermo Scientific) according to the manufacturer’s instructions, and visualized via autoradiography. To ensure adequate sample had been loaded for antibody detection, membranes were stripped and re-probed with anti-β-actin primary antibody (sc47778, Santa Cruz Biotechnology, Dallas, Texas, USA) and anti-mouse IgG secondary antibody (NA9310V, GE Healthcare).

### Statistical analyses

Statistical analyses were performed with GraphPad Prism 9 software. Study group variances were calculated using Welch’s ANOVA test for subject age and Chi-square tests for sex and race. For odds ratios (OR), 95% confidence intervals (CI) were computed using the Woolf logit method, and *P* values were calculated using Fisher’s exact tests. For all statistical analyses, the predetermined α level was 0.05. All reported *P* values are two-sided.

## Results

### Patient characteristics

In this pilot case–control study, we compared the prevalence of gastric herpesvirus infections in patients with FGIDs, with and without comorbid FM, to that in controls. Thirty patients with one or more FGIDs and comorbid FM, 15 patients with one or more FGIDs, and 15 control patients were enrolled. The most prevalent FGIDs among case patients were IBS, functional dyspepsia, functional dysphagia, and functional gallbladder disorder (see Table 1, Patient demographics). Other FGIDs among case patients included chronic nausea vomiting syndrome, functional heartburn, reflux hypersensitivity, functional abdominal bloating/distention, centrally mediated abdominal pain syndrome, and unspecified functional bowel disorder. See Supplementary Table 1 for individual participant data.

**Table 1:** Patient demographics

	Group 1 FGID + FM *n* (%)	Group 2 FGID-only *n* (%)	Group 3 Control *n* (%)
Total number of subjects per group	30	15	15
Demographic characteristics			
Mean years of age at biopsy (± SD)	45.1 (± 12.4)	61.1 (± 8.1)	56.9 (± 8.9)
Sex			
Male	3 (10)	9 (60)	9 (60)
Female	27 (90)	6 (40)	6 (40)
Race			
White/Caucasian	23 (76.7)	8 (53.3)	6 (40.0)
Black/African American	7 (23.3)	7 (46.7)	9 (60.0)
FGIDs (*n*, %)			
Irritable bowel syndrome	29 (96.7)	7 (46.7)	0
IBS-C^a^	10 (33.3)	3 (20.0)	0
IBS-D^a^	5 (16.7)	2 (13.3)	0
IBS-M^a^	14 (46.7)	2 (13.3)	0
Functional dyspepsia	5 (16.7)	9 (50.0)	0
Functional dysphagia	10 (33.3)	1 (6.7)	0
Functional gallbladder disorder	11 (36.7)	1 (6.7)	0
Chronic nausea vomiting syndrome	2 (6.7)	1 (6.7)	0
Functional heartburn	0	1 (6.7)	0
Reflux hypersensitivity	0	1 (6.7)	0
Functional abdominal bloating/distention	0	1 (6.7)	0
Unspecified functional bowel disorder	0	1 (6.7)	0
Centrally mediated abdominal pain syndrome	0	1 (6.7)	0

^a^Irritable bowel syndrome subtypes: IBS-C = predominantly constipation, IBS-D = predominantly diarrhea, IBS-M = alternating diarrhea and constipation

### Gastric HSV-1 infection is associated with FGIDs

**Fig. 1:**
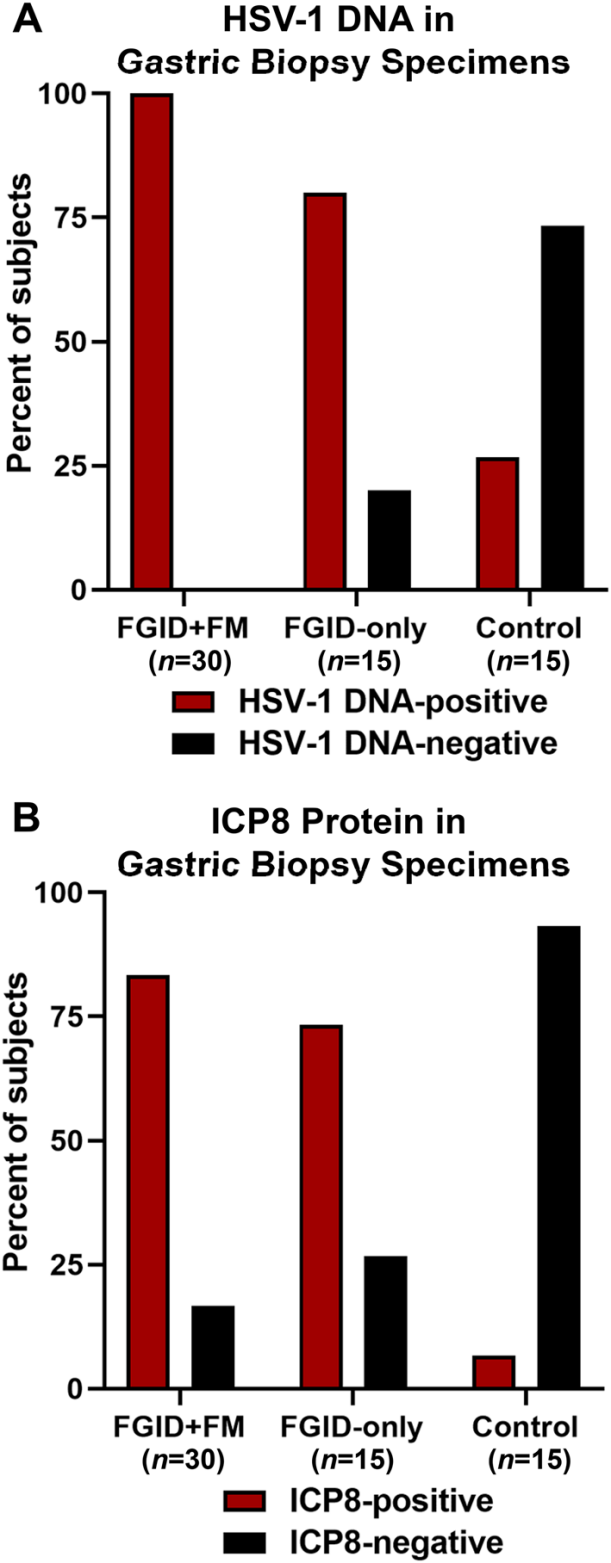
Prevalence of HSV-1 infection in the gastric mucosa of case and control patients. Percentages of gastric biopsy specimens from case and control patients that were positive/negative for HSV-1 DNA as indicated by PCR and DNA sequencing (**A**) and ICP8 protein as determined by immunoblotting (**B**) are shown

To determine whether gastric herpesvirus infections were positively associated with FGIDs, gastric biopsy specimens were examined for the presence of herpesvirus DNA and ICP8 protein. DNA isolated from gastric biopsy tissue was used as the template for nested PCR reactions with primers that allow the amplification of a region of the DNA polymerase gene from any herpesvirus, as noted above [57]. Following PCR amplification, herpesvirus DNA was detected in the biopsies of 100% (30/30) of Group 1 patients (with FGIDs and comorbid FM), 80% (12/15) of Group 2 patients, and 26.7% (4/15) of control patients (Fig. 1A). Following DNA sequencing of the PCR products, NCBI BLASTN alignments and careful examination of the sequencing trace reports indicated HSV-1 was the only herpesvirus present in any of the PCR products. The presence of HSV-1 DNA in gastric biopsy specimens was significantly associated with FGIDs both in the presence and absence of comorbid FM (see Table 2, Association of FGIDs with HSV-1 DNA, ICP8, gastritis, and *Helicobacter pylori* infection).

**Table 2:** Association of FGIDs with HSV-1 DNA, ICP8, gastritis, and *Helicobacter pylori* infection in gastric mucosa biopsies

	Group 1 FGID + FM *n* = 30	Group 2 FGID-only *n* = 15
Association of FGIDs with HSV-1 DNA		
HSV-1 DNA-positive, *n*	30	12
Odds ratio	155.90	11.00
95% CI	7.77–3129.00	2.00–60.57
*P* value	< 0.001	0.009
Association of FGIDs with ICP8 protein		
HSV-1 ICP8-positive, *n*	25	11
Odds ratio	70.00	38.50
95% CI	7.42–660.50	3.75–395.40
*P* value	< 0.001	< 0.001
Association of FGIDs with histologically identified gastritis		
Gastritis-positive, *n*	21	15
Odds ratio	0.85	12.13
95% CI	0.21–3.39	0.59–248.50
*P* value	> 0.999	0.100
Association of FGIDs with *H. pylori* infection		
*H. pylori*-positive, *n*	4	2
Odds ratio	0.42	0.4231
95% CI	0.09–2.00	0.06–2.77
*P* value	0.410	0.651

*CI* confidence interval, *FGID* functional gastrointestinal disorder, *FM* fibromyalgia, *HSV*-1 herpes simplex virus type 1, *H. pylori* *Helicobacter pylori*

Due to the sensitivity of nested PCR, it is possible the identification of HSV-1 DNA in gastric biopsies might have resulted from detection of latent viral genomes and/or virions passing through the GI tract at the time of biopsy collection. Therefore, biopsy specimens were also examined via immunoblotting using an antibody specific to the ICP8 protein. ICP8 is an HSV-1 protein involved in viral genome replication that is produced in cells during lytic (replicating), but not latent, infections. Although required for HSV-1 replication, ICP8 is not packaged into virions. Thus, this nonstructural HSV-1 protein serves as a marker to differentiate cell-associated lytic infection from latent infection and/or free virus passing through the GI tract. ICP8 was detected in the biopsies of 83.3% (25/30) of Group 1 patients, 73.3% (11/15) of Group 2 patients, and 6.7% (1/15) of control patients (Fig. 1B). The presence of ICP8 was significantly associated with FGIDs both in the presence and absence of comorbid FM (see Table 2). Across all groups, ICP8 presence was also significantly associated with IBS (OR 5.44, 95% CI 1.73–17.17; *P* = .005).

### Histologically identified gastritis and *Helicobacter pylori* infection are not associated with FGIDs

**Fig. 2:**
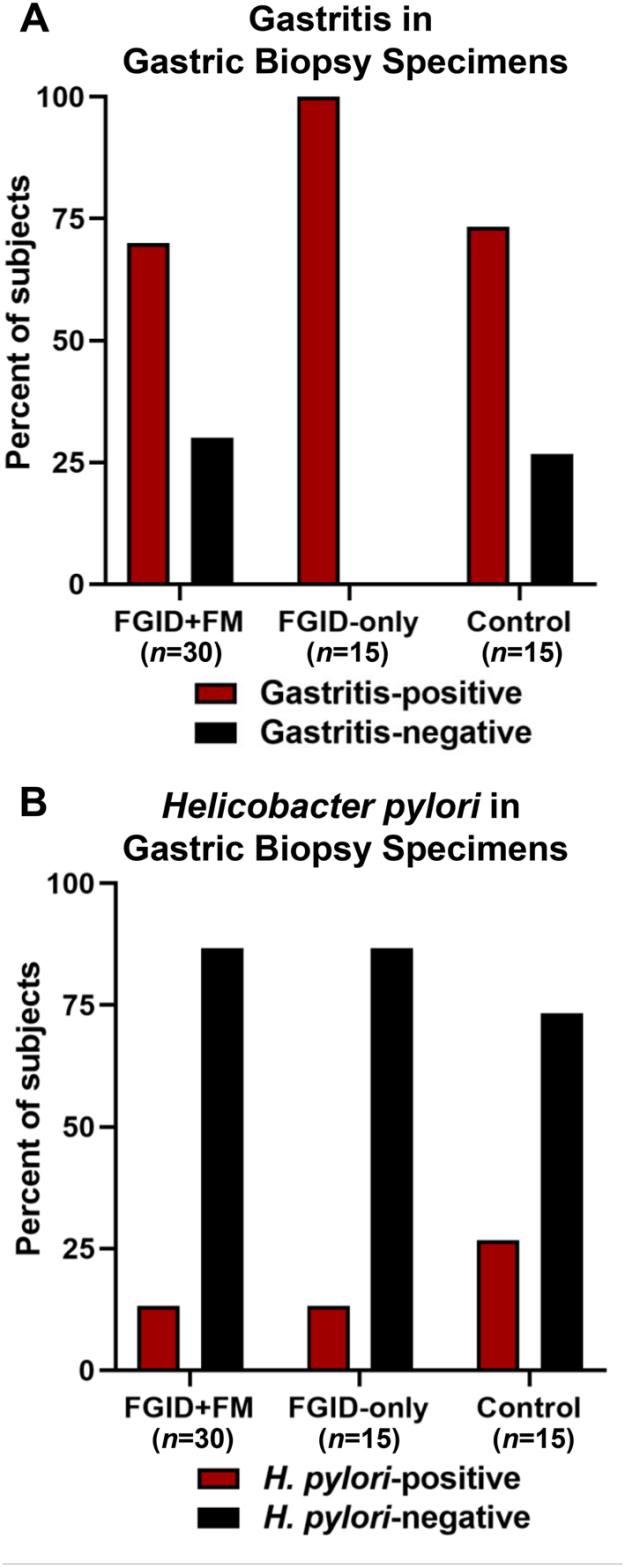
Prevalence of histologically identified gastritis and *Helicobacter pylori* infection in case and control patients. Percentages of gastric biopsy specimens from case and control patients that were positive/negative for histological gastritis (**A**) and *H. pylori* infection (**B**) as determined by clinical pathologists are shown

Histological examination was performed by clinical pathologists to identify gastritis at the microscopic level. Histologically identified gastritis was observed in the biopsy specimens of 70% (21/30) of Group 1 patients, 100% (15/15) of Group 2 patients, and 73.3% (11/15) of control patients (Fig. 2A). Thus, histologically identified gastritis was seen broadly in both case and control patients and was not significantly associated with HSV-1 infection or FGIDs (see Table 2).

*Helicobacter pylori* has been proposed to contribute to the symptoms of IBS, dyspepsia, dysphagia, and other FGIDs [58–63]. To examine possible associations of *H. pylori* infection with FGIDs, Giemsa-stained gastric biopsy specimens were examined by clinical pathologists. *H. pylori* was identified in the biopsy specimens of 13.3% (4/30) of Group 1 patients, 13.3% (2/15) of Group 2 patients, and 26.7% (4/15) of control patients (Fig. 2B). Thus, *H. pylori* was much less prevalent than HSV-1 in our study population and was not significantly associated with FGIDs (see Table 2). Coinfection with *H. pylori* and HSV-1 was identified in 6.7% (2/30) of Group 1 patients, 13.3% (2/15) of Group 2 patients, and 0 control patients.

## Discussion

In this pilot case–control study, we found a significant association between gastric HSV-1 infection, as indicated by the presence of both HSV-1 DNA and ICP8 protein, and FGIDs in the presence and absence of comorbid FM. The presence of the nonstructural viral protein ICP8 in gastric mucosa specimens indicates HSV-1 identified in the biopsied regions was undergoing active replication at the time of tissue collection. Although several herpesviruses are known to infect the gastric mucosa [24–34], HSV-1 was the only herpesvirus detected in the gastric biopsy specimens from our patient population. Careful examination of sequencing trace reports showed none of the other 8 human herpesviruses were present in PCR products derived from the gastric biopsies examined for this study. As a neurotropic herpesvirus, HSV-1 invades, establishes latency within, and spreads between neurons of the central, peripheral, and enteric nervous systems [35–37, 40–44]. The ability of HSV-1 to spread trans-synaptically through complex neural pathways is relevant when considering a potential role for this virus in FGIDs and overlapping upper and lower GI symptoms.

Proper gut function largely depends on both the extrinsic and intrinsic neural pathways that innervate the GI tract. Extrinsic neural pathways are composed of both sympathetic and parasympathetic projections, as well as visceral afferents. Intrinsic neural pathways are provided by the enteric nervous system (ENS), which is embedded in the gut wall and controls functions such as gut motility, digestive secretion and absorption, blood flow, and facets of the local immune system. The ENS contains thousands of linked small ganglia that form the myenteric and submucosal plexuses. Ganglia within these plexuses are connected via internodal strands that carry axons considerable distances and enable the rapid conduction of neuronal signals along the bowel.

Animal studies have shown HSV-1 can infect and establish latency in intrinsic neurons of the myenteric and submucosal ganglia and extrinsic neurons of the nodose and dorsal root ganglia, which project to the gut [35, 37, 44, 45]. Human cadaver and surgical case studies have reported latent HSV-1 in nodose, dorsal root, and celiac ganglionic neurons, which innervate the alimentary canal from the pharynx to the colon [36, 40, 41, 43]. Following HSV-1 reactivation, which can occur more frequently than previously thought [64, 65], concomitant lytic replication within the innervated gut mucosal epithelium can result in enteric disorders such as herpetic gastritis, herpes esophagitis, and gastric ulcers [24, 26, 27, 66, 67]. Latency and frequent reactivation of HSV-1 within GI tract-innervating neurons could impart an etiologic mechanism for the chronic nature of FGIDs. In addition, the ability of this virus to spread trans-synaptically through the complex neural pathways that regulate the gut provides a potential common pathogenesis mechanism for overlapping upper and lower GI symptoms. Indeed, HSV-1 spread through gut-brain neural pathways following intragastric infection of animal models resulted in intestinal neuromuscular dysfunction, intestinal dysmotility, and immune-mediated enteric neuronal damage [37, 42, 45, 68]. Future research aimed at furthering our understanding of the interplay between HSV-1 infection and gut-brain neural pathways will provide important insights into the role this virus may play in FGIDs and overlapping upper and lower GI symptoms.

In the present study, neither FGIDs nor gastric HSV-1 infection was associated with histologically identified gastritis. The literature contains conflicting reports on the association of *H. pylori* infection with FGIDs [58–63, 69–71]. In our study population, *H. pylori* was more prevalent in control patients than in case patients and was not associated with FGIDs. Although it was beyond the scope and hypothesis of this study to examine all potentially relevant microbial species, it is well established that gut microbial dysbiosis can affect GI health. Thus, future studies may identify microbes that contribute to the etiology of FGIDs alone or in concert with HSV-1.

FGIDs are often comorbid with FM and other functional somatic syndromes [48–50]. We previously showed that a combination therapy aimed at preventing herpesvirus reactivation and lytic replication was safe and efficacious in the treatment of FM symptoms [47]. In the current study, we found gastric HSV-1 infection, as indicated by the presence of HSV-1 DNA and ICP8 protein in gastric mucosa biopsies, was significantly associated with FGIDs both in the presence and absence of comorbid FM. The authors know of no prior publications evaluating the association of HSV-1 and FGIDS.

Limitations of this pilot study include its small sample size, group demographic differences, single site selection biases, and a lack of corroborating serological data. The small sample size of this study likely contributed to the unusually large odds ratios and broad 95% CIs computed for our data set. As the control subjects were presenting for endoscopic examinations for reasons other than FGIDs or FM, they were demographically distinct from the FGID population. Despite these limitations, the strong association between HSV-1 and FGIDs found herein indicates larger, multi-site studies are warranted. If a common pathogenesis is responsible for FGID and FM symptoms in some patients, HSV-1 may comprise a viable etiologic candidate and therapeutic target.

## Conclusions

In the current study, gastric HSV-1 infection, as indicated by the presence of both HSV-1 DNA and ICP8 protein, was associated with FGID diagnoses in the presence and absence of comorbid FM. Despite the limitations of this pilot study, the strong associations found indicate larger studies examining the role of HSV-1 in FGIDs are warranted. In addition, studies evaluating the efficacy of antiviral therapeutic approaches to the treatment of FGID symptoms may result in improved outcomes for this sizeable patient population.

## Supplementary Information

Below is the link to the electronic supplementary material.

Supplementary file1 (DOCX 26KB)

## Data Availability

The blinded datasets obtained during this study are available from the corresponding author upon reasonable request.
