# Supplementary material for: Gastric herpes simplex virus type 1 infection is associated with functional gastrointestinal disorders in the presence and absence of comorbid fibromyalgia: a pilot case–control study
Source: Infection. 2022 Apr 21;50(5):1303–11. doi: 10.1007/s15010-022-01823-w (PMC9522778; doi:10.1007/s15010-022-01823-w)
Supplement: Supplementary file 1 — Supplementary file1 (DOCX 26KB) [file 15010_2022_1823_MOESM1_ESM.docx]

Article Title: Gastric Herpes Simplex Virus Type 1 Infection is Associated with Functional Gastrointestinal Disorders in the Presence and Absence of Comorbid Fibromyalgia: A Pilot Case-Control Study

Journal Name: Infection

Authors: Carol Duffy, William L. Pridgen, and Richard J. Whitley

Corresponding Author: Carol Duffy, The University of Alabama, [cduffy3@ua.edu](mailto:cduffy3@ua.edu)

Supplementary Table 1. Sex and Age of Study Participants with Associated Rome IV FGIDs, Histological Findings, HSV-1 DNA Status, HSV-1 ICP8 Status, and *Helicobacter pylori* Status.

|  | Sex | Age | Rome IV diagnosis* | Histological Findings | HSV-1 DNA | HSV-1 ICP8 | | *H. pylori* | |
| --- | --- | --- | --- | --- | --- | --- | --- | --- | --- |
| Case Group 1 (FGID + FM) | | | | | | | | | |
| 1 | Male | 52 | C1 (IBS-M), E1a | Mild chronic gastritis | + | | + | | - |
| 2 | Female | 26 | C1 (IBS-M), E1a | Reactive gastropathy, no gastritis | + | | + | | - |
| 3 | Female | 43 | C1 (IBS-M), B3a | Mild chronic gastritis | + | | - | | - |
| 4 | Female | 60 | C1 (IBS-M), E1a | No significant pathologic changes | + | | - | | - |
| 5 | Female | 19 | C1 (IBS-D) | Mild chronic gastritis | + | | + | | - |
| 6 | Female | 59 | C1 (IBS- C), E1a | Mild chronic gastritis | + | | + | | - |
| 7 | Female | 50 | C1 (IBS-C), E1a | Chronic gastritis | + | | + | | - |
| 8 | Female | 51 | C1 (IBS-M), B1 | Moderate edema with mucosal congestion | + | | + | | - |
| 9 | Female | 43 | C1 (IBS-C), B1 | Chronic follicular gastritis | + | | + | | + |
| 10 | Female | 29 | C1 (IBS-M), E1a, B1 | Mild chronic gastritis | + | | + | | - |
| 11 | Female | 59 | C1 (IBS-M) | Mild chronic gastritis | + | | + | | - |
| 12 | Female | 56 | C1 (IBS-C), A5, E1a | Mild chronic gastritis | + | | + | | + |
| 13 | Female | 21 | C1 (IBS-M) | Mild chronic gastritis | + | | + | | - |
| 14 | Female | 62 | C1 (IBS- M), B1 | Mild chronic gastritis | + | | + | | - |
| 15 | Female | 41 | C1 (IBS- D), A5 | Acute erosive gastritis | + | | + | | - |
| 16 | Male | 44 | C1 (IBS-D) | Reactive gastropathy, no gastritis | + | | + | | - |
| 17 | Female | 49 | C1 (IBS-C), A5, E1a | Chronic gastritis | + | | + | | - |
| 18 | Female | 57 | C1 (IBS-C), A5 | Mild chronic gastritis | + | | - | | + |
| 19 | Female | 48 | C1 (IBS- M), B1 | Reactive gastropathy, no gastritis | + | | + | | - |
| 20 | Female | 22 | C1 (IBS-C) | Chronic gastritis | + | | + | | - |
| 21 | Male | 51 | C1 (IBS-C), E1a, A5 | Reactive gastropathy, no gastritis | + | | + | | - |
| 22 | Female | 41 | C1 (IBS-C) | Mild chronic gastritis | + | | - | | - |
| 23 | Female | 36 | C1 (IBS-M), A5 | Reactive gastropathy, no gastritis | + | | + | | - |
| 24 | Female | 58 | B3a, E1a | No significant pathologic changes | + | | + | | - |
| 25 | Female | 42 | C1 (IBS-M), A5 | Mild chronic gastritis | + | | + | | - |
| 26 | Female | 46 | C1 (IBS-C), A5 | Mild chronic gastritis | + | | + | | - |
| 27 | Female | 51 | C1 (IBS-M), A5 | Reactive gastropathy, no gastritis | + | | + | | - |
| 28 | Female | 30 | C1 (IBS-M), E1a, A5 | Chronic gastritis | + | | - | | + |
| 29 | Female | 35 | C1 (IBS-D) | Mild chronic gastritis | + | | + | | - |
| 30 | Female | 64 | C1 (IBS-D) | Mild chronic gastritis | + | | + | | - |
| Case Group 2 (FGID-only) | | | | | | | | | |
| 1 | Male | 63 | C5 | Mild chronic gastritis | + | + | | - | |
| 2 | Female | 51 | C1 (IBS-C) | Mild chronic gastritis | + | + | | - | |
| 3 | Male | 59 | D1 | Mild chronic gastritis | + | + | | - | |
| 4 | Male | 74 | B1 | Active chronic gastritis | + | + | | + | |
| 5 | Male | 56 | C4, B1 | Mild chronic gastritis | + | + | | - | |
| 6 | Male | 70 | B1 | Chronic gastritis | + | + | | - | |
| 7 | Female | 61 | C1 (IBS-D), A2), A3, B1 | Mild chronic gastritis | + | + | | - | |
| 8 | Male | 52 | C1 (IBS-C), B1, E1a | Mild chronic gastritis | + | - | | - | |
| 9 | Male | 50 | B1 | Chronic follicular gastritis | + | + | | + | |
| 10 | Male | 70 | B1 | Chronic follicular gastritis | + | + | | - | |
| 11 | Female | 51 | C1 (IBS-D) | Mild chronic gastritis | + | + | | - | |
| 12 | Female | 61 | C1 (IBS-C), B1 | Mild chronic gastritis | + | + | | - | |
| 13 | Female | 64 | C1 (IBS-M) | Mild chronic gastritis | - | - | | - | |
| 14 | Female | 57 | C1 (IBS-M), B3a, B1 | Mild chronic gastritis | - | - | | - | |
| 15 | Male | 73 | A5 | Mild chronic gastritis | - | - | | - | |
| Control Group | | | | | | | | | |
| 1 | Male | 65 | None | Mild chronic gastritis | + | + | | - | |
| 2 | Female | 53 | None | No significant pathologic changes | + | - | | - | |
| 3 | Female | 53 | None | Mild chronic gastritis | + | - | | - | |
| 4 | Male | 59 | None | Follicular gastritis | - | - | | + | |
| 5 | Male | 60 | None | Active chronic gastritis | - | - | | + | |
| 6 | Male | 67 | None | Chronic follicular gastritis | - | - | | - | |
| 7 | Female | 58 | None | Mild chronic gastritis | - | - | | - | |
| 8 | Female | 79 | None | Mild chronic gastritis | - | - | | - | |
| 9 | Male | 60 | None | Active chronic gastritis | - | - | | + | |
| 10 | Female | 63 | None | Reactive gastropathy, no gastritis | - | - | | - | |
| 11 | Male | 45 | None | Mild chronic gastritis | - | - | | - | |
| 12 | Male | 45 | None | Reactive gastropathy, no gastritis | - | - | | - | |
| 13 | Male | 49 | None | Mild chronic gastritis | + | - | | - | |
| 14 | Male | 64 | None | Reactive gastropathy, no gastritis | - | - | | - | |
| 15 | Female | 45 | None | Moderate chronic gastritis | - | - | | + | |

FGID = functional gastrointestinal disorder; FM = fibromyalgia; *H. pylori* = *Helicobacter pylori*; HSV-1 = herpes simplex virus type 1; ICP8 = infected cell protein 8

*Rome IV diagnosis key: A2=functional heartburn; A3=reflux hypersensitivity; A5=functional dysphagia; B1=functional dyspepsia; B3a=chronic nausea vomiting syndrome; C1=irritable bowel syndrome, with subtypes C=predominantly constipation, D=predominantly diarrhea, M=alternating diarrhea and constipation; C4=functional abdominal bloating/distention; C5=unspecified functional bowel disorder; D1=centrally mediated abdominal pain syndrome; E1a=functional gallbladder disorder
